# Organic Dust Exposure Enhances SARS-CoV-2 Entry in a PKC*α*- and ADAM-17-Dependent Manner

**DOI:** 10.3390/ijtm4030032

**Published:** 2024

**Authors:** Abenaya Muralidharan, Christopher D. Bauer, Claire G. Nissen, St Patrick Reid, Jill A. Poole, Todd A. Wyatt

**Affiliations:** 1 Department of Pathology and Microbiology, College of Medicine, University of Nebraska Medical Center, Omaha, NE 68198, USA;; 2 Department of Internal Medicine, College of Medicine, University of Nebraska Medical Center, Omaha, NE 68198, USA;; 3 Department of Environmental, Agricultural & Occupational Health, College of Public Health, University of Nebraska Medical Center, Omaha, NE 68198, USA;; 4 Veterans Affairs Nebraska-Western Iowa Health Care System, Omaha, NE 68105, USA

**Keywords:** SARS-CoV-2, ACE2, organic dust (ODE), PKCα, ADAM-17

## Abstract

SARS-CoV-2, the causative agent of the COVID-19 pandemic, has had a global impact, affecting millions over the last three years. Pre-existing lung diseases adversely affect the prognosis of infected COVID-19 patients, and agricultural workers routinely exposed to inhalable organic dusts have substantial increased risk for developing chronic lung diseases. In previous studies, we characterized the protein kinase C (PKC)-dependent airway inflammation mediated by organic dust extract (ODE) derived from dust collected from swine confinement facilities in in vitro and in vivo models. Here, we studied the effect of ODE on SARS-CoV-2 pseudoviral infection in mice and human bronchial epithelial cells (BEAS-2B). In wild-type (WT) and transgenic mice expressing the human angiotensin I-converting enzyme 2 (ACE2) receptor (SARS-CoV-2 entry receptor), ODE increased ACE2 shedding by ADAM-17 in the lungs. After repeated ODE treatments, the increased soluble ACE2 correlated to higher pseudovirus titer in the mouse lungs. In the human bronchial epithelial cells, ODE augmented PKCα activity in WT cells, and membrane ACE2 expression was diminished in PKCα-dominant negative cells. Unlike in the mice, increasing membrane ACE2 levels by treating with PKCα or ADAM-17 inhibitors and a low dose of ODE enhanced pseudoviral entry in vitro. Following viral entry, IL-8 secretion by the cells was diminished in a PKCα- and ADAM- 17-independent manner. Together, the complex mechanisms involved in the synergistic effects of agricultural dust and SARS-CoV-2 highlight the importance of studying dust-mediated changes to immunity against circulating pathogens.

## 1. Introduction

The coronavirus disease 2019 (COVID-19) pandemic caused by severe acute respiratory syndrome coronavirus 2 (SARS-CoV-2) has been one of the largest global pandemics in history. Due to its high transmission rate, SARS-CoV-2 spread to over 216 countries and territories in less than 8 months. As of September 2022, more than 610 million confirmed cases and over 6.5 million deaths have been reported worldwide [1]. Although most individuals are asymptomatic following infection, some individuals will develop a range of symptoms including fever, pneumonia, acute respiratory distress, multiorgan failure, and death. The severity of symptoms and resulting prognosis depend heavily on pre-existing health conditions and exposure to risk factors [2]. COVID-19-associated morbidity and mortality markedly increase in the presence of comorbidities such as diabetes and liver, kidney, cardiovascular, and pulmonary disease [3].

The inhalation of bioaerosols for long periods of time can lead to the development of lung diseases that predispose individuals to severe COVID-19. Large, confined animal feeding operations in the United States produce bioaerosols that pose a significant health risk to agricultural workers. Long-term exposures to these aerosols can have deleterious effects on pulmonary functions, increasing the risk of respiratory diseases including asthma, chronic bronchitis, chronic obstructive pulmonary disease, hypersensitivity pneumonitis, and pulmonary fibrosis [4]. These agricultural workers often report respiratory and flu-like symptoms mainly due to the highly inflammatory composition of the aerosols [5–7].

Organic dust collected from swine operations is complex and composed of Gram-positive and Gram-negative bacteria, fungal spores, and other particulates that can elicit pro-inflammatory responses in the lungs [8]. Indeed, exposure to complex swine confinement organic dust extracts (ODEs) has been shown to induce an influx of neutrophils, macrophages, and lymphocytes, as well as the release of pro-inflammatory cytokines resulting in airway hyper-responsiveness in animal models, resembling human disease [9]. Cytokines, such as tumor necrosis factor (TNF)-α and interleukin (IL-6), and neutrophil- attracting chemokines, such as murine CXCL1 and CXCL2 or human IL-8, are released following ODE exposure in animals and humans [9].

In our previous studies, we demonstrated that ODE activates protein kinase C (PKC), mediating the release of IL-6 and IL-8 from human bronchial epithelial cells, a process shown to be dependent on TNF-α and TNF receptor [10,11]. In addition, ODE-induced lung inflammatory outcomes are dependent upon the Toll-like receptor (TLR)/myeloid differentiation primary response 88 (MyD88) signaling pathways [12]. It has also been recognized that signals, including the activation of PKC and TLRs responding to pathogens, can activate ADAM-17 and its “sheddase” activity [13]. A disintegrin and metalloprotease 17 (ADAM-17), also called TACE (tumor necrosis factor-α-converting enzyme), is a membrane protease that, when activated, removes the ectodomains of membrane proteins like angiotensin-converting enzyme 2 (ACE2), an entry receptor for SARS-CoV-2. ADAM-17 is expressed in many tissues such as muscle, thymus, heart, small intestine, gonads, placenta, kidney, pancreas, and lung [14]. ACE2 is a type I transmembrane carboxymonopeptidase protein expressed in several organs ubiquitously, with the highest levels of expression in the cardiovascular system, brain, testicles, kidneys, intestine, and lungs [15]. During infection, the SARS-CoV-2 spike protein binds the ACE2 receptor on target host cells, allowing the fusion of the viral envelope and host-cell membrane, leading to subsequent viral entry [16,17].

Here, since ODE alters ADAM-17 function through PKC signaling, which can then affect ACE2, we studied the changes in SARS-CoV-2 entry following ODE exposure and its dependence on PKCα and ADAM-17 in vivo in mice and in vitro in a human bronchial epithelial cell line.

## 2. Materials and Methods

### 2.1. SARS-CoV-2 Pseudovirus

To generate SARS-CoV-2 pseudovirus, 293T cells (ATCC: CRL-3216) grown in Dulbecco’s Modified Eagle Medium (DMEM) with 10% fetal bovine serum were transfected with 7 μg of pLVx-tdTomato-N1 Vector (Takara Bio, Palo Alto, CA, USA) and one tube of Lenti-XTM SARS-CoV-2 Packaging Mix (WT Spike, Full Length) (Takara Bio, Palo Alto, CA, USA) according to the manufacturer’s instructions. The supernatant from the cells containing pseudovirus was harvested 48 h post-transfection, centrifuged, and stored at −80 °C. The supernatant was sucrose-purified using ultracentrifugation for animal experiments and stored at −80 °C.

### 2.2. Mice Experiments

Eight-week-old wild-type (WT) or transgenic mice expressing the human ACE2 receptor (Jackson Labs, Bar Harbor, ME USA) were used for all animal experiments. WT mice were intranasally instilled with saline or 12.5% organic dust extract (ODE; characterized in Boissy [18]) once (single instillation) or 13 times over 3 weeks (repetitive instillation). Lungs were then collected following the final ODE exposure and homogenized, and the murine ACE2 levels were quantified. Similarly, humanized ACE2 mice were intranasally treated with sterile saline, 12.5% ODE, or TAPI-1 (1 μM, Sigma-Aldrich, St. Louis, MO, USA) and 12.5% ODE. After ODE exposure, lungs were collected, homogenized, and human ACE2 was quantified. For pseudovirus infection experiments, humanized ACE2 mice were intranasally treated once or 13 times with ODE and infected with purified pseudovirus. Five days post-infection, lungs were collected for homogenization and RNA extraction to determine viral titer. All animal experiments were reviewed and approved by the Institutional Animal Care and Use Committee of University of Nebraska Medical Center (Protocol #22-070-11-FC) and were conducted in accordance with the Institutional Animal Care and Use Committee guidelines and regulations.

### 2.3. Mouse Lung ACE2 Quantitation

Cell-free soluble ACE2 in the supernatant of homogenized and centrifuged mouse lungs was determined using a Mouse ELISA kit in the WT mice and a Human ELISA kit in the humanized ACE2 mice according to the manufacturer’s (R&D Systems, Minneapolis, MN, USA) instructions. ACE2 is reported as pg/mL.

### 2.4. RNA Extraction and Quantitative Polymerase Chain Reaction (qPCR)

RNA was isolated from the mouse lungs using Direct-zol^™^ RNA Miniprep Plus (Zymo Research, Irvine, CA, USA). Lungs were collected in TRI Reagent^®^ (Zymo Research, Irvine, CA, USA), homogenized, and centrifuged. The cell-free supernatant was then used for RNA extraction according to the manufacturer’s instructions.

The virus titer was determined using the Lenti-XTM qRT-PCR Titration Kit (Takara Bio, Palo Alto, CA, USA) according to the manufacturer’s instructions. The Lenti-XTM RNA Control Template was used alongside the samples to quantify RNA copies. A QuantStudio 3 Real-Time PCR machine (Applied Biosystems, Waltham, MA, USA) was used with QuantStudio Design and Analysis software version 1.5.1 (Applied Biosystems, Waltham, MA, USA) for analysis. The results are expressed as RNA copies per lung (log).

### 2.5. BEAS-2B Cells

Wild-type (WT) and PKCα-dominant negative (DN) human bronchial epithelial cell line (BEAS-2B) cells were generated and cultured as previously described [19].

### 2.6. PKCα Activity Assay

WT and PKCα-DN BEAS-2B cells were treated with 0.5% and 5% ODE or media for 1 h at 37 °C and 5% CO_2_. Calcium-dependent PKC alpha isoform-specific activity was measured using radiolabeled substrate phosphate transfer of P32-ATP as previously described [19].

### 2.7. BEAS-2B Cell Treatments and Infection

At 80–90% confluency, in a 96-well format, WT and PKCα-DN BEAS-2B cells were treated with the PKCα inhibitor Gö 6976 (1 μM; Sigma-Aldrich), ADAM17 (TACE) inhibitor TAPI-1 (20 μM), or media for 1 h at 37 °C, and the cells were subsequently washed to remove treatments, and stimulated with and without 0.5% ODE. Cytotoxicity was observed with higher concentrations of HDE in response to pseudovirus. After incubating at 37 °C for 1 h, cells were either collected for ACE2 expression by flow cytometry analyses or subsequently stimulated with unpurified SARS-CoV-2 pseudovirus. The virus was diluted in cell growth media containing 6 μg/mL Polybrene (Millipore-Sigma, Darmstadt, Germany). Subsequently,18 to 24 h later, cells were washed to remove SARS-CoV-2 pseudovirus and vehicle control, and fresh media was applied without stimulants for an additional 24 h (48 h from the time of the addition of the virus). Cell-free supernatant was collected to quantify IL-8 production. The cells were fixed for pseudovirus quantification by immunofluorescence.

### 2.8. ACE2 Expression by Flow Cytometry

Following ODE stimulation, WT and PKCα-DN BEAS-2B cells pre-treated with/without the PKCα or ADAM17 inhibitor were trypsinized and processed for cell surface ACE2 expression. Cells were washed and stained with monoclonal mouse IgG_2A_ anti-human antibody against ACE2 (Alexa Fluor 647, Clone 535919, R&D Systems) for 30 min on ice. The stained cells were washed twice and fixed with 4% paraformaldehyde (Thermo Fisher Scientific, Waltham, MA, USA). Cells were acquired on a NovoCyte flow cytometer (ACEA Biosciences, San Diego, CA, USA). Unstained controls were used for gating. Post-acquisition, data were exported and analyzed using NovoExpress 1.5.0 (Agilent Technologies, Santa Clara, CA, USA) software.

### 2.9. Quantification of BEAS-2B Pseudovirus Infection by Immunofluorescence

Treated and infected BEAS-2B cells were fixed with 4% paraformaldehyde (Thermo Fisher Scientific, Waltham, MA, USA) for 10 min at room temperature. The fixative was then removed and Hoechst 33342 nuclear stain (Invitrogen, Waltham, MA, USA) was added at 1/20,000 dilution for 15 min at room temperature. Stained cells were visualized using the Operetta CLS^™^ system (Perkin Elmer, Waltham, MA, USA). dTomato-positive cells (infected with pseudovirus) were identified under 20× air objective and 9 fields/well were analyzed using Harmony 4.9 (Perkin Elmer, Waltham, MA, USA) software.

### 2.10. Interleukin-8 (IL-8) ELISA

Epithelial cell-free supernatants were quantitated for human IL-8/CXCL8 using the DuoSet ELISA kit (R&D Systems) according to manufacturer’s instructions. IL-8 is reported as ng/mL.

### 2.11. Statistical Analysis

Data are presented as the mean ± standard error of mean (SEM) with scatter plots depicted for each data point. Student’s *t*-test was used to assess statistical differences between two groups, and one-way analysis of variance (ANOVA) or two-way ANOVA was used to assess statistical differences among three or more experimental groups with Tukey’s post hoc test for multiple comparisons between any two groups. Statistical significance was accepted at a *p* value < 0.05. All statistical analyses were performed using GraphPad Prism 9 (San Diego, CA, USA) software.

## 3. Results

### 3.1. ODE Single and Repetitive Exposure Increases Murine Lung Soluble ACE2 Levels Dependent upon ADAM-17 with an Associated Effect on SARS-CoV-2 Pseudovirus Infectivity

**Figure 1.**
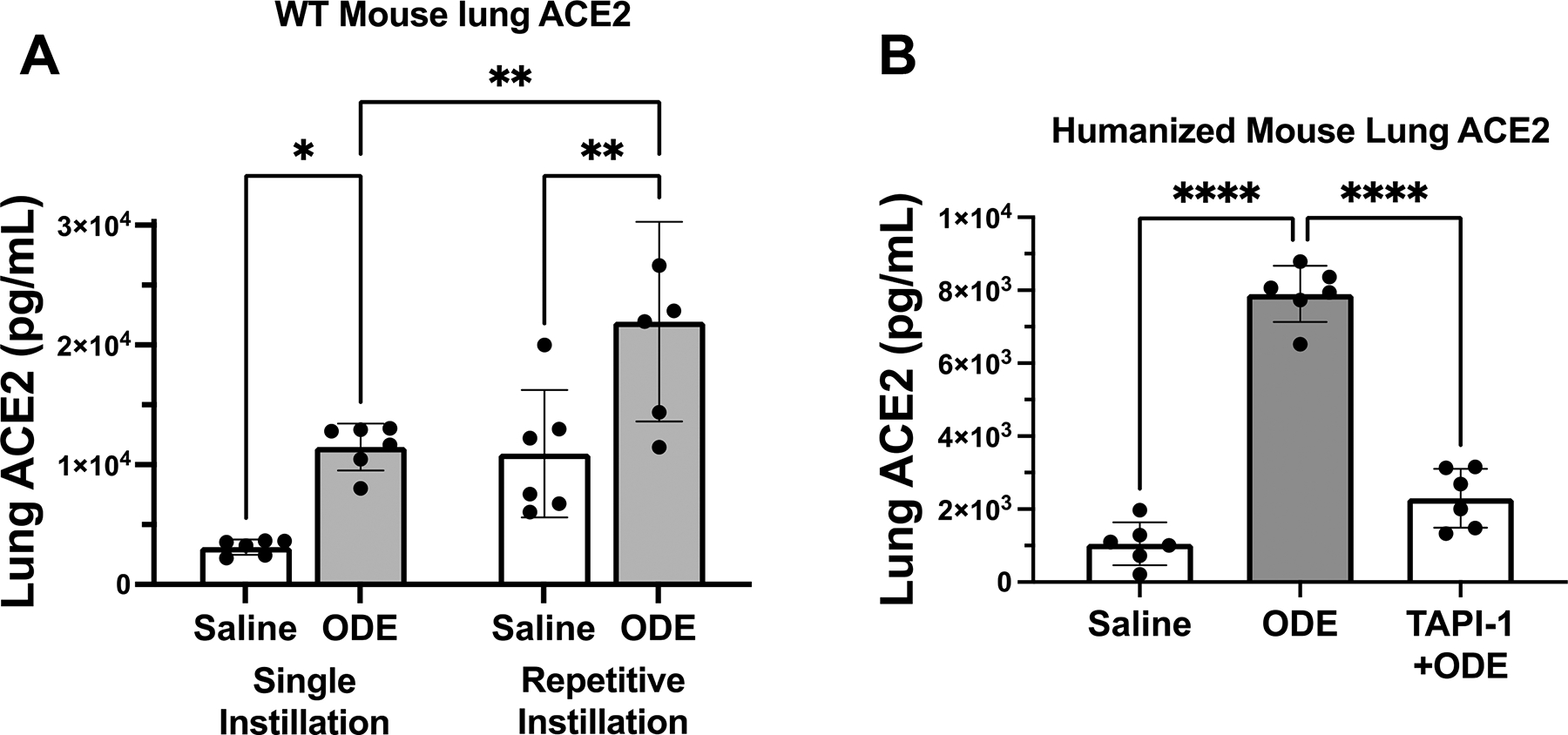

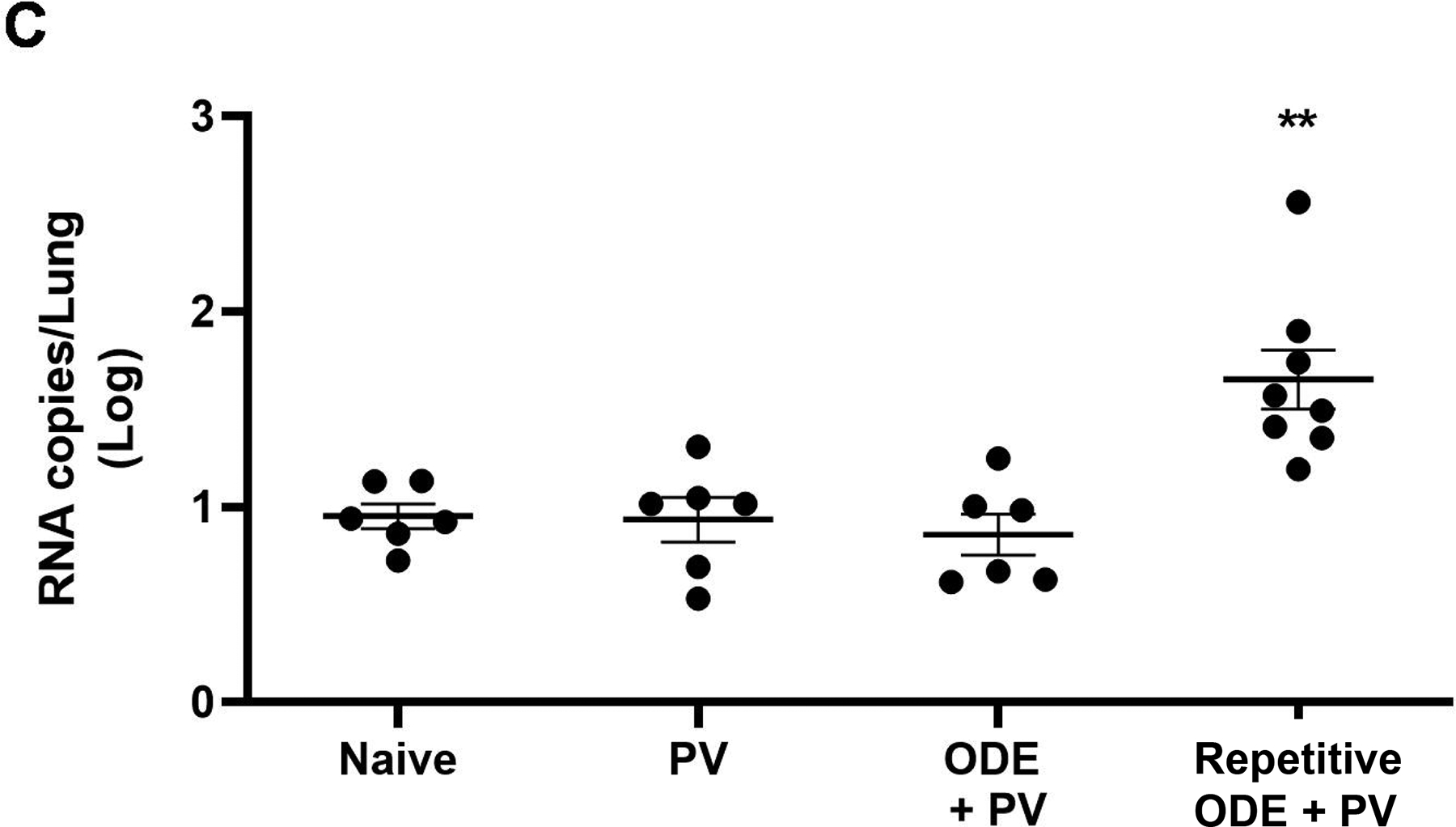
ODE single and repetitive exposure increases murine lung soluble ACE levels dependent upon ADAM-17 with associated effect on SARS-CoV-2 pseudovirus infectivity. Scatter plots with bars depict mean with SEM of lung ACE2 levels of (**A**) wild-type (WT) mice following single and repetitive (13 times) ODE exposures and (**B**) humanized ACE2 mice treated with or without TAPI- 1, an ADAM-17 inhibitor, prior to single instillation with saline or 12.5% ODE exposure (*n* = 6 mice/group). Humanized ACE2 mice were exposed to a single dose or repetitive doses of ODE prior to SARS-CoV-2 pseudovirus (PV) infection with lungs collected 5 days post-infection. (**C**) Scatter plot with mean and SEM depicted viral titer determined by qPCR (*n* = 6–8 mice/group). * *p* < 0.05, ** *p* < 0.01, **** *p* < 0.0001; groups compared using Student’s *t*-test in (**A**) and one-way ANOVA with Tukey’s post hoc test in (**B**,**C**).

First, we aimed to determine whether lung levels of ACE2, a SARS-CoV-2 entry receptor, were shed from the cell membrane following the administration of ODE in mice. Indeed, a one-time and repetitive ODE treatment(s) significantly increased soluble ACE2 levels as compared to saline control, and moreover, repetitive ODE treatment for 3 weeks resulted in further ACE2 levels versus a single ODE exposure (Figure 1A). The ODE- induced increase in soluble ACE2 levels was also confirmed in the humanized ACE2 transgenic mice whereby ODE treatment significantly increased lung ACE2 levels, and this response was strikingly reduced with treatment with the ADAM-17 inhibitor TAPI-1 (Figure 1B). These findings demonstrate that ODE-induced lung shed ACE2 levels are dependent upon ADAM-17 activity. This ODE-mediated increase was reversed by the addition of TAPI-1, an ADAM-17 inhibitor, reiterating the role of ADAM-17 in cleaving ACE2 from the cell membrane. Corresponding to increased lung soluble ACE2 levels induced by ODE, there was an increase in SARS-CoV-2 pseudovirus entry in the lungs of humanized ACE2 mice pre-treated with repetitive but not a one-time ODE exposure (Figure 1C). The ex vivo pseudoviral infection of mouse tracheal epithelial cells collected from humanized ACE2 mice following 0.5% ODE treatment also resulted in a higher viral titer compared to untreated cells. Together, ODE enhanced ADAM-17-dependent ACE2 shedding in mice, which was associated with increasing SARS-CoV-2 spike protein-mediated viral entry following repeated ODE exposure.

### 3.2. ODE Treatment Increases Human Bronchial Epithelial Cell PKCα Activity

**Figure 2.**
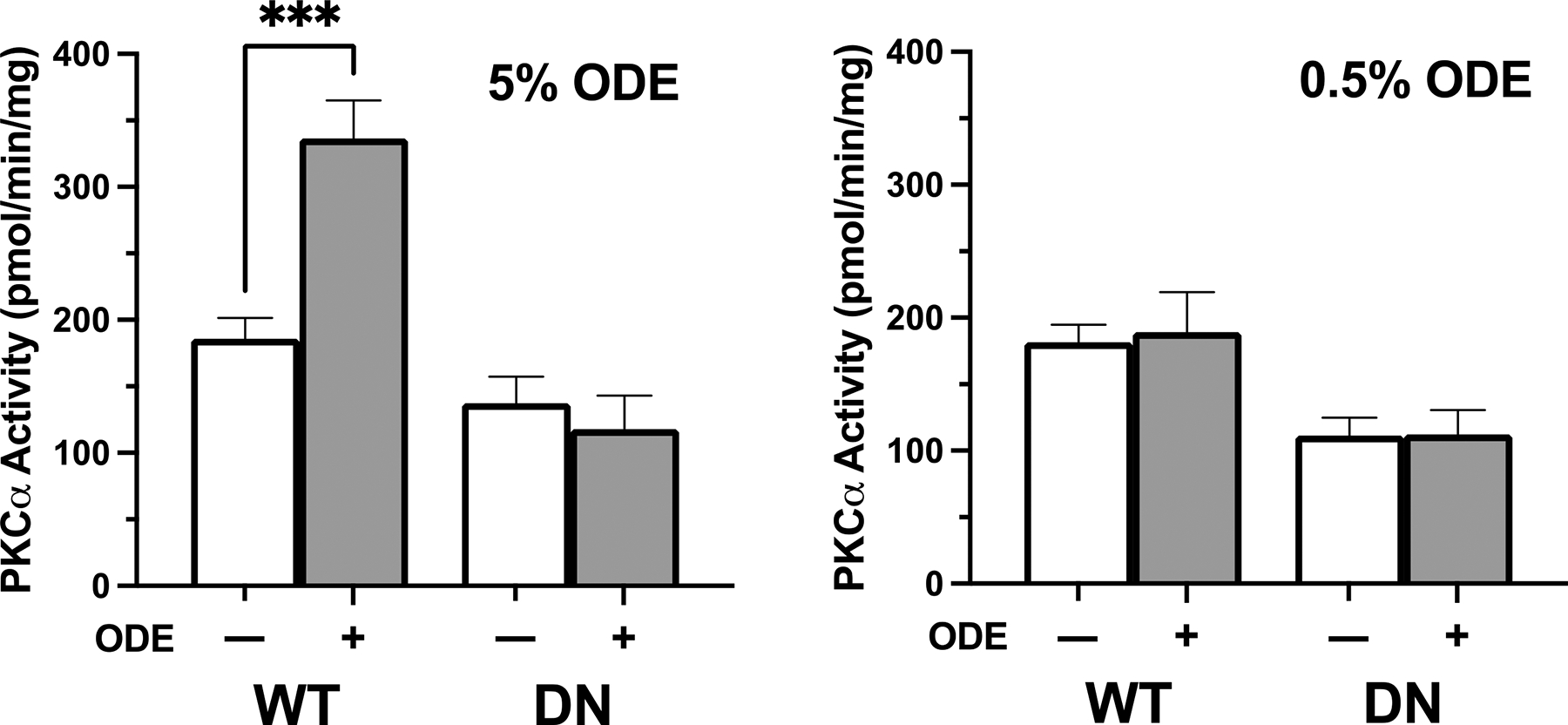
ODE-induced human bronchial epithelial cell PKCα activity is abrogated in PKCα-deficient cells. Wild-type (WT) and PKCα-deficient (DN) BEAS-2B cells were exposed to 0.5 or 5% ODE for 1 h in vitro. Following the treatment, PKCα activity was measured. WT cells treated with 5% ODE had significantly higher PKC activity than all other groups. Data shown are mean ± SEM; *n* = 6 per experiment; experiments were repeated 3 times; *** *p* < 0.001 (two-way ANOVA with Tukey’s post hoc test).

PKCα is recognized to activate ADAM-17 [20], and here we sought to determine whether ODE induced PKCα activity in a human cell line in vitro. Wild-type (WT) and PKCα-dominant negative (DN) BEAS-2B cells, an immortalized epithelial cell line from human bronchial epithelium, were treated with 0.5 and 5% ODE. Following 1 h of treatment, PKCα activity was quantified in the WT and DN cells. DN cells had minimal PKCα activity that did not change with ODE treatment whereas WT cells had a significant increase in PKCα activity when exposed to 5% ODE (Figure 2). However, no activation of PKCα was observed in response to 0.5% ODE in either WT or DN cells. Therefore, in the presence of functional PKCα, ODE enhanced its activity, which could then promote ADAM-17-mediated ACE2 shedding (Figures 1 and 2).

### 3.3. Inhibition of PKCα and ADAM-17 along with ODE Treatment Synergistically Increases Membrane ACE2 Levels, Enhancing SARS-CoV-2 Pseudovirus Entry in BEAS-2B Cells In Vitro

**Figure 3.**
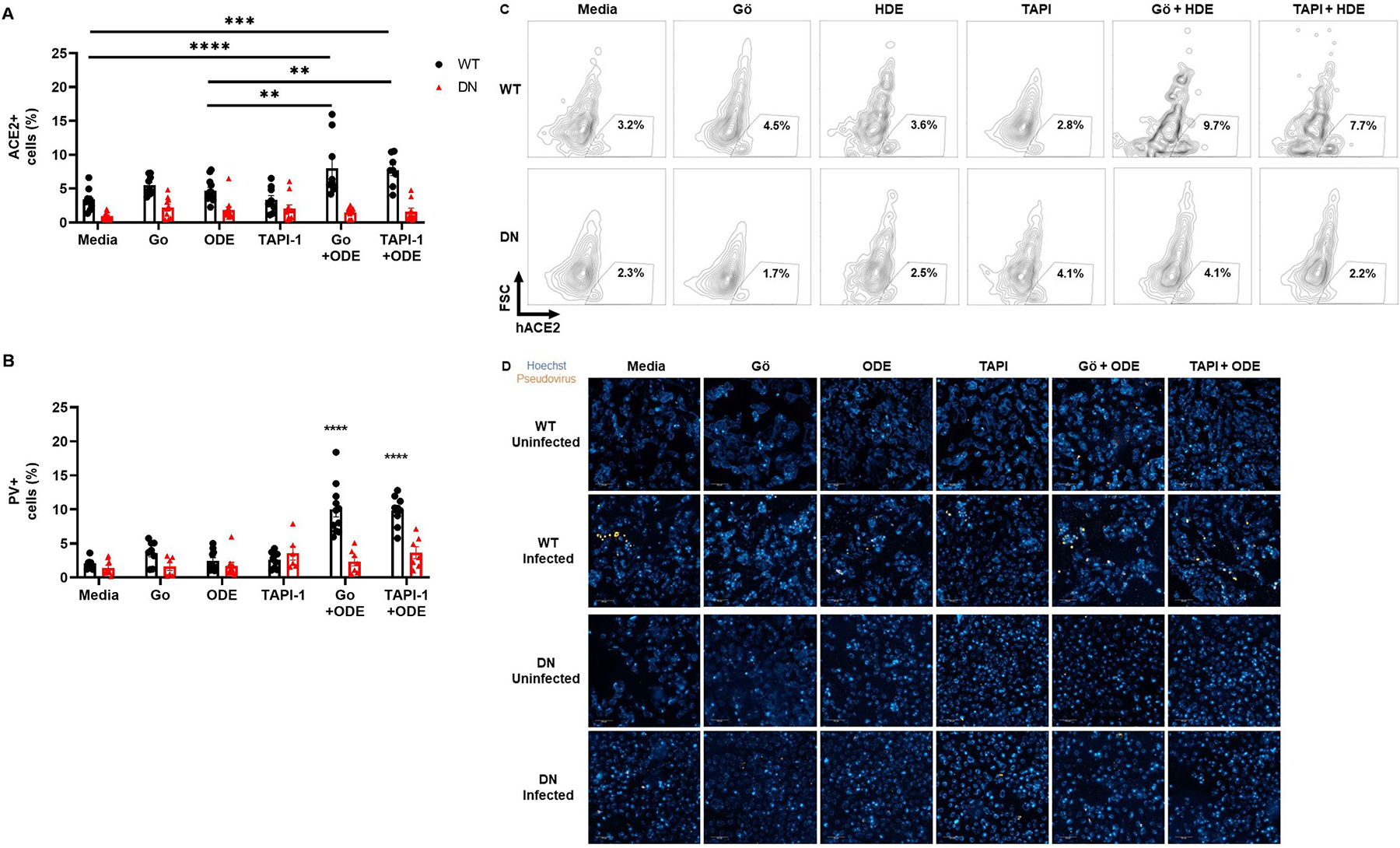
Inhibition of PKCα or ADAM-17 along with ODE treatment synergistically increases membrane ACE2 levels enhancing SARS-CoV-2 pseudovirus entry in BEAS-2B cells in vitro. (**A**) Wild-type (WT) and PKCα-deficient (DN) BEAS-2B cells were treated with Gö 6976 (a PKCα inhibitor), TAPI-1 (an ADAM-17 inhibitor), 0.5% ODE, or a combination for 1 h in vitro. The cells were then collected and stained for flow cytometry analysis of membrane ACE2 expression. (**B**) Treated cells were infected for 48 h with SARS-CoV-2 pseudovirus expressing fluorescent dTomato. The cells were then fixed, stained with Hoechst nuclear stain, and analyzed using Operetta CLS. WT cells treated with both ODE and inhibitor had significantly higher infection than single-treated WT groups. (**C**) Representative flow cytometry images showing ACE2 gating. (**D**) Representative immunofluorescence images from Operetta CLS showing pseudovirus-infected cells (20× magnification; scale bar: 100 μm). Data shown are mean ± SEM; *n* = 9 per group; experiments were repeated 4 times; ** *p* < 0.01, *** *p* < 0.001, **** *p* < 0.0001 (two-way ANOVA with Tukey’s post hoc test).

To determine the functional importance of PKCα and ADAM-17 to SARS-CoV-2 pseudovirus entry in the setting ODE exposure, the generated PKCα WT and dominant negative BEAS-2B cells were treated with ODE in the presence/absence of Gö 6976 (PKCα inhibitor) and TAPI-1 (ADAM-17 inhibitor) for 1 h. First, we confirmed that 5% ODE induced PKCα that was abrogated in the PKCα-dominant negative cells (Figure 2). Next, there was no increase in cell surface membrane ACE2 expression with treatment with ODE, Gö 6976, or TAPI-1 alone in PKCα WT cells; however, ACE2 expression increased in the combination treatment of Gö 6976 + ODE and TAPI-1 + ODE versus control (Figure 3A,C). Furthermore, there was a higher infectivity rate by SARS-CoV-2 pseudovirus in PKCα WT cells treated with Gö 6976 + ODE as well as TAPI-1 + ODE (Figure 3B,D).

Notably, ODE treatment alone did not result in higher membrane ACE2 expression or pseudovirus entry compared to the untreated group. This could be because we used sub-stimulatory doses of ODE (0.5%) for these BEAS-2B treatment/infection experiments to ensure that cell viability was not affected due to ODE during the 48 h pseudovirus infection. There was no evidence of cytotoxicity as there were no differences in the levels of lactate dehydrogenase among all treatment groups. Together, PKCα and ADAM-17 signaling help prevent SARS-CoV-2 spike protein-mediated viral entry into cells in vitro by decreasing ACE2 expression on the cell membrane.

### 3.4. SARS-CoV-2 Pseudovirus Infection Reduces IL-8 Secretion in a PKCα-Independent Manner in Low-Dose ODE-Treated BEAS-2B Cells

**Figure 4.**
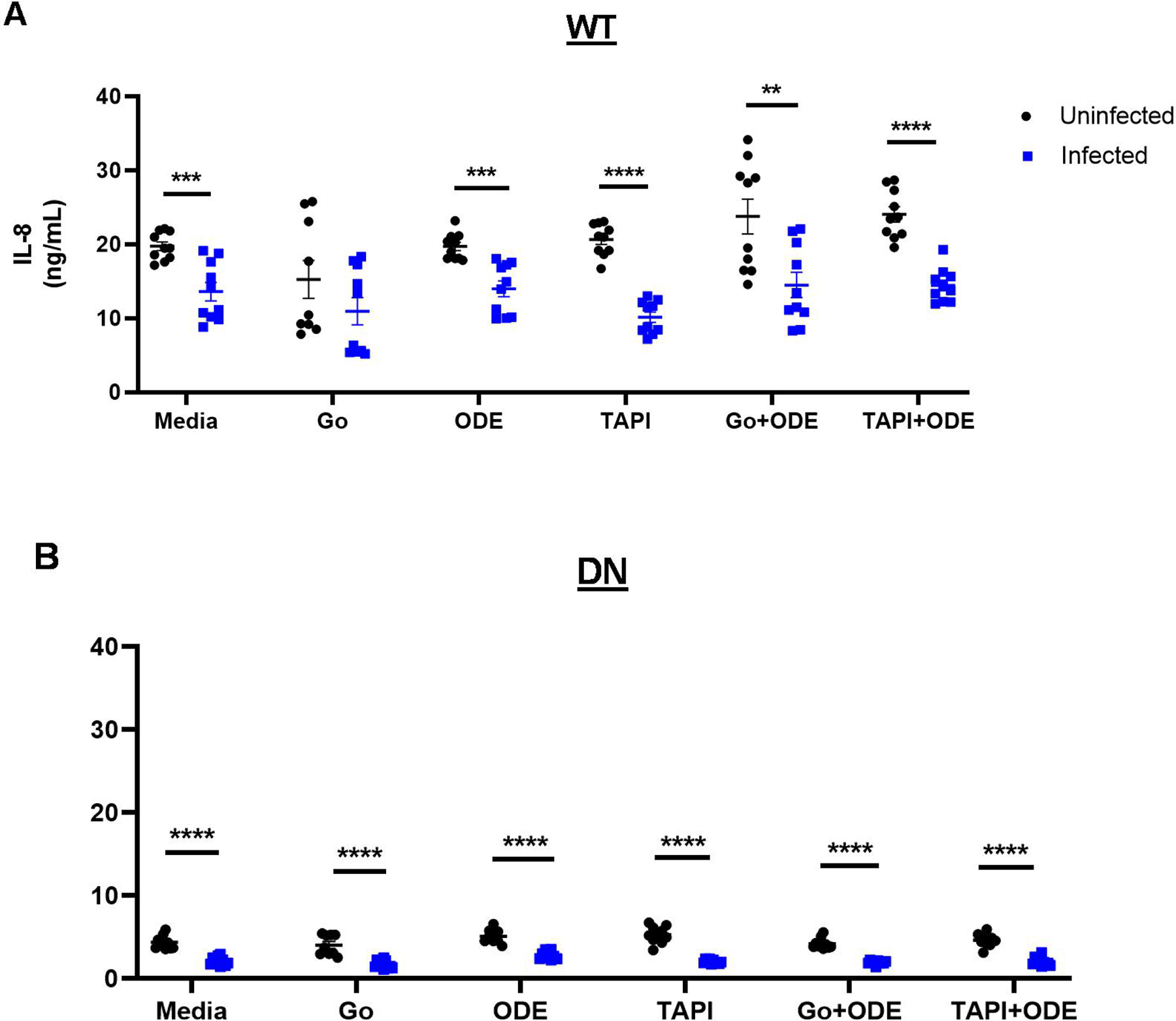
Infection with SARS-CoV-2 pseudovirus reduces IL-8 secretion in a PKCα- and ADAM-17-independent manner in 0.5% ODE-treated cells. (**A**) Wild-type (WT) and (**B**) PKCα-deficient (DN) BEAS-2B cells were treated with Gö 6976 (a PKCα inhibitor), TAPI-1 (an ADAM-17 inhibitor), 0.5% ODE, or a combination for 1 h in vitro and infected with SARS-CoV-2 pseudovirus for 48 h. The supernatant was then collected for IL-8 measurement using ELISA. Data shown are mean ± SEM; *n* = 9 per group; experiments were repeated 2 times; ** *p* < 0.01, *** *p* < 0.001, **** *p* < 0.0001 (Student’s *t*-test).

**Figure 5.**
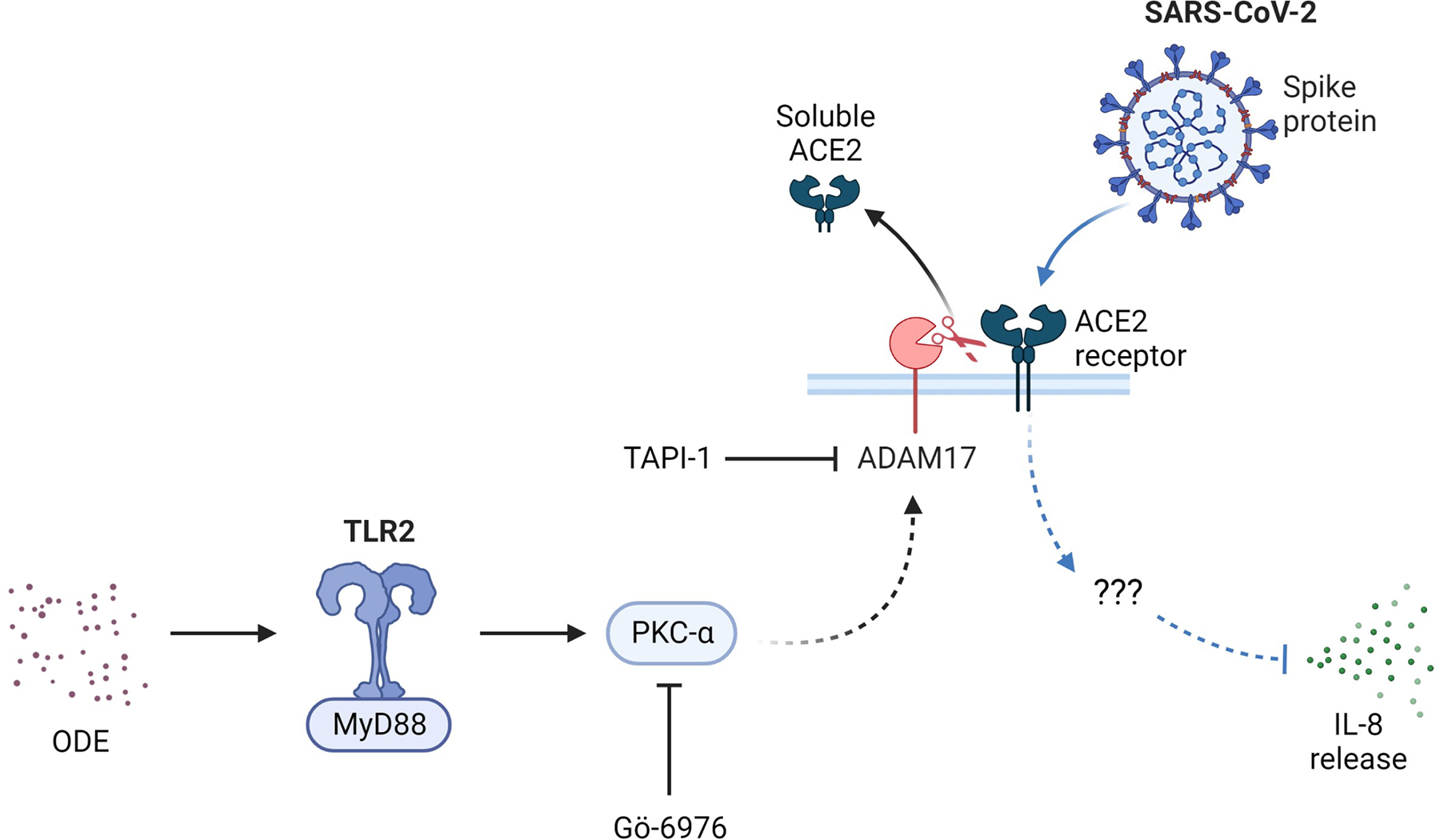
Proposed mechanism through which agricultural dust exposure could affect SARS-CoV-2 entry in vitro (created with BioRender.com). Organic dust exposure (ODE) activates Toll-like receptor 2 (TLR2) and MyD88, which then activates protein kinase C alpha (PKCα). Through intermediates, PKCα activates ADAM-17 which cleaves the ACE2 receptor on the cell membrane producing soluble ACE2. PKCα can be inhibited by the addition of Gö 6976 and ADAM-17 can be inhibited by the addition of TAPI-1. If membrane ACE2 is intact, upon SARS-CoV-2 infection, the viral spike protein can bind the receptor and through unknown intermediates diminish IL-8 release in vitro.

Following a 48 h exposure to SARS-CoV-2 pseudovirus in the presence of Gö 6976, TAPI-1, and/or ODE in WT and DN BEAS-2B cells, we quantified cytokine levels in the supernatant media. Because lower concentrations of ODE were used to prevent cell death, neither the treatments nor the pseudovirus had any effect on PKCα-mediated IL-6 secretion, as expected in BEAS-2B cells. However, IL-8 secretion was significantly decreased after pseudovirus infection in both WT and DN cells (Figure 4A,B). The overall quantity of IL-8 detected in DN cells was much lower than in WT cells, emphasizing the importance of PKCα signaling for IL-8 production (Figure 4B). Interestingly, IL-8 secretion was not altered by the inhibition of PKCα or ADAM-17 or by ODE (Figure 4A,B). Therefore, engaging membrane ACE2 by the SARS-CoV-2 spike protein diminishes IL-8 secretion in a PKCα- and ADAM-17-independent manner in vitro (Figure 5).

## 4. Discussion

Agricultural workers are exposed to a wide range of respiratory irritants every day they work. These aerosolized irritants have complex compositions that can trigger the immune system in a variety of ways. Long-term exposure to such triggers can significantly deteriorate workers’ respiratory health by not only predisposing them to chronic lung diseases, but also having a serious impact on their immunity against respiratory infections. During a global pandemic caused by a respiratory virus, these essential agricultural workers put themselves at considerable risk. Indeed, studies conducted during the early stages of the COVID-19 pandemic showed a high prevalence of SARS-CoV-2 among agricultural field workers, with about half the workers testing positive [21–23]. Therefore, it is crucial to understand the mechanisms underlying dust-mediated changes to immune responses against pathogens, especially SARS-CoV-2.

In this study, we focused on exposure to dust collected from swine confinement facilities (ODE) in the United States. In our previous works, the mechanisms underlying ODE-mediated airway inflammation have been well characterized in in vitro and in vivo models [24]. We have shown the sequential activation of PKCα leading to pro-inflammatory cytokine and chemokine release in response to the ODE treatment of bronchial epithelial cells in vitro [10]. In vivo, mice exposed to a single dose of ODE had pronounced neutrophil influx and high levels of inflammatory mediators in bronchoalveolar lavage fluid. Interestingly, repeated ODE exposure exacerbated lung histopathology even though inflammatory cytokine levels and cell influx were not as striking as single exposure [25]. In this study, ODE treatment resulted in higher levels of soluble ACE2 in mouse lungs in both wild-type and humanized ACE mice in an ADAM-17-dependent manner, with repeated treatment further increasing ACE2 shedding in wild-type mice (Figure 1A,B). This increase in soluble ACE2 directly correlated to SARS-CoV-2 pseudovirus entry in the repeated-exposure model in humanized ACE mice (Figure 1C).

However, in vitro, ODE-mediated upregulation of membrane ACE2 following PKCα and ADAM-17 inhibition was important for increased SARS-CoV-2 entry (Figure 3). This suggests that, in a human bronchial epithelial cell line (BEAS-2B), PKCα and ADAM-17 signaling induced by ODE help prevent SARS-CoV-2 spike protein-mediated viral entry into cells by decreasing ACE2 expression on the cell membrane. This points to the limitations associated with the in vitro model of ODE exposure. Apart from the direct ACE2 receptor and viral spike protein interaction, soluble ACE2 can also facilitate SARS-CoV-2 entry through receptor-mediated endocytosis [26] as seen in the mouse model (Figure 1). Changes in cell culture conditions, such as temperature, can lower the rate of receptor-mediated endocytosis, which can then affect the infection efficiency of the cells. However, the use of transgenic in vitro models does allow for better understanding of the mechanisms involved.

The PKCα-deficient (DN) BEAS-2B cells used in this study had significantly lower levels of membrane ACE2 in untreated and treated samples compared to WT cells, suggesting that functional PKCα may play a role in ACE2 expression (Figure 3A). Even though low membrane ACE2 correlated to low pseudovirus infection in the DN cells (Figure 3B), suggesting a protective role, infection with pathogenic SARS-CoV-2 would have resulted in severe pathology in the absence of ACE2. Indeed, studies with influenza and respiratory syncytial virus have shown that administering recombinant ACE2 had beneficial effects on virus-induced lung lesions [27,28].

The downregulation of membrane ACE2 by SARS-CoV-2 can also lead to pulmonary injury as severe as lethal lung failure [29–31]. The high expression of ACE2 in the lungs may, in fact, be related to the protective effects the receptor provides [32]. Indeed, ACE2 protected mice infected with SARS-CoV-1 from acute lung disease since spike protein binding reduced ACE2 surface expression due to the internalization of the ACE2–virus complex [33]. Similarly, the SARS-CoV-2-mediated downregulation of ACE2 also decreases the protective effects of ACE2 in the kidneys, heart, gut, and lungs [34–36].

In addition, ACE2-mediated SARS-CoV-2 entry may have an anti-inflammatory effect. In BEAS-2B cells, SARS-CoV-2 pseudovirus infection significantly diminished IL-8 release under all treatment conditions in a PKCα- and ADAM-17-independent manner (Figure 4). IL-8, a neutrophil chemoattractant, plays a key role in inducing inflammation following ODE exposure [37]. In our previous studies, we showed that ODE activates PKC, which can then activate ADAM-17, mediating the release of IL-6 and IL-8 in a TNF-α- and TNF receptor-dependent pathway in bronchial epithelial cells [10,11,13]. The activation of ADAM-17 can subsequently induce ACE2 receptor shedding from the cell membrane (Figure 5). On the other hand, ADAM-17 activation also affects pro-inflammatory responses mediated by IL-8 and anti-inflammatory responses by TNF receptor shedding [13]. Thus, a virus-induced reduction in pro-inflammatory responses elicited by ODE may delay immune cell recruitment, impeding viral clearance and prolonging infection. Studies are ongoing to determine the mechanisms underlying the inhibition of IL-8 production by membrane ACE2 activation.

Although short exposures to low doses of ODE could create a pro-inflammatory lung environment that may help enhance viral clearance, long-term ODE exposure-induced inflammatory responses may predispose individuals to severe COVID-19. Compounded with the respiratory conditions associated with chronic ODE exposure, increased viral entry into cells through soluble ACE2 and delayed viral clearance due to the virus-mediated suppression of inflammatory responses may lead to a persistent infection, further exacerbating pulmonary disease in agricultural workers.

## Data Availability

The original contributions presented in the study are included in the article; further inquiries can be directed to the corresponding author.

## References

[1] Ganesh B; Rajakumar T; Malathi M; Manikandan N; Nagaraj J; Santhakumar A; Elangovan A; Malik YS Epidemiology and pathobiology of SARS-CoV-2 (COVID-19) in comparison with SARS, MERS: An updated overview of current knowledge and future perspectives. Clin. Epidemiol. Glob. Health 2021, 10, 100694. 33462564 10.1016/j.cegh.2020.100694 PMC7806455

[2] Guan WJ; Ni ZY; Hu Y; Liang WH; Ou CQ; He JX; Liu L; Shan H; Lei CL; Hui DSC; Clinical Characteristics of Coronavirus Disease 2019 in China. N. Engl. J. Med 2020, 382, 1708–1720. 32109013 10.1056/NEJMoa2002032 PMC7092819

[3] Huang C; Wang Y; Li X; Ren L; Zhao J; Hu Y; Zhang L; Fan G; Xu J; Gu X; Clinical features of patients infected with 2019 novel coronavirus in Wuhan, China. Lancet 2020, 395, 497–506. 31986264 10.1016/S0140-6736(20)30183-5 PMC7159299

[4] Sigsgaard T; Basinas I; Doekes G; de Blay F; Folletti I; Heederik D; Lipinska-Ojrzanowska A; Nowak D; Olivieri M; Quirce S; Respiratory diseases and allergy in farmers working with livestock: A EAACI position paper. Clin. Transl. Allergy 2020, 10, 29. 32642058 10.1186/s13601-020-00334-x PMC7336421

[5] Bongers P; Houthuijs D; Remijn B; Brouwer R; Biersteker K Lung function and respiratory symptoms in pig farmers. Br. J. Ind. Med 1987, 44, 819–823. 3689717 10.1136/oem.44.12.819 PMC1007926

[6] Haglind P; Rylander R Occupational exposure and lung function measurements among workers in swine confinement buildings. J. Occup. Med 1987, 29, 904–907. 3681501

[7] Holness DL; O’Blenis EL; Sass-Kortsak A; Pilger C; Nethercott JR Respiratory effects and dust exposures in hog confinement farming. Am. J. Ind. Med 1987, 11, 571–580. 3591805 10.1002/ajim.4700110509

[8] Yang X; Haleem N; Osabutey A; Cen Z; Albert KL; Autenrieth D Particulate Matter in Swine Barns: A Comprehensive Review. Atmosphere 2022, 13, 490.

[9] Warren KJ; Dickinson JD; Nelson AJ; Wyatt TA; Romberger DJ; Poole JA Ovalbumin-sensitized mice have altered airway inflammation to agriculture organic dust. Respir. Res 2019, 20, 51. 30845921 10.1186/s12931-019-1015-0 PMC6407255

[10] Wyatt TA; Slager RE; Heires AJ; Devasure JM; Vonessen SG; Poole JA; Romberger DJ Sequential activation of protein kinase C isoforms by organic dust is mediated by tumor necrosis factor. Am. J. Respir. Cell Mol. Biol 2010, 42, 706–715. 19635931 10.1165/rcmb.2009-0065OC PMC2891498

[11] Burvall K; Palmberg L; Larsson K Expression of TNFalpha and its receptors R1 and R2 in human alveolar epithelial cells exposed to organic dust and the effects of 8-bromo-cAMP and protein kinase A modulation. Inflamm. Res 2005, 54, 281–288. 16134057 10.1007/s00011-005-1356-7

[12] Bauer C; Kielian T; Wyatt TA; Romberger DJ; West WW; Gleason AM; Poole JA Myeloid differentiation factor 88- dependent signaling is critical for acute organic dust-induced airway inflammation in mice. Am. J. Respir. Cell Mol. Biol 2013, 48, 781–789. 23492189 10.1165/rcmb.2012-0479OC PMC3727869

[13] Zipeto D; Palmeira JDF; Arganaraz GA; Arganaraz ER ACE2/ADAM17/TMPRSS2 Interplay May Be the Main Risk Factor for COVID-19. Front. Immunol 2020, 11, 576745. 33117379 10.3389/fimmu.2020.576745 PMC7575774

[14] Gooz M ADAM-17: The enzyme that does it all. Crit. Rev. Biochem. Mol. Biol 2010, 45, 146–169. 20184396 10.3109/10409231003628015 PMC2841225

[15] Hamming I; Timens W; Bulthuis ML; Lely AT; Navis G; van Goor H Tissue distribution of ACE2 protein, the functional receptor for SARS coronavirus. A first step in understanding SARS pathogenesis. J. Pathol 2004, 203, 631–637. 15141377 10.1002/path.1570 PMC7167720

[16] Kuhn JH; Li W; Choe H; Farzan M Angiotensin-converting enzyme 2: A functional receptor for SARS coronavirus. Cell. Mol. Life Sci 2004, 61, 2738–2743. 15549175 10.1007/s00018-004-4242-5 PMC7079798

[17] Hofmann H; Pohlmann S Cellular entry of the SARS coronavirus. Trends Microbiol. 2004, 12, 466–472. 15381196 10.1016/j.tim.2004.08.008 PMC7119031

[18] Boissy RJ; Romberger DJ; Roughead WA; Weissenburger-Moser L; Poole JA; LeVan TD Shotgun pyrosequencing metagenomic analyses of dusts from swine confinement and grain facilities. PLoS ONE 2014, 9, e95578. 24748147 10.1371/journal.pone.0095578 PMC3991671

[19] Allen-Gipson DS; Floreani AA; Heires AJ; Sanderson SD; MacDonald RG; Wyatt TA Cigarette smoke extract increases C5a receptor expression in human bronchial epithelial cells. J. Pharmacol. Exp. Ther 2005, 314, 476–482. 15843499 10.1124/jpet.104.079822

[20] Hahn D; Pischitzis A; Roesmann S; Hansen MK; Leuenberger B; Luginbuehl U; Sterchi EE Phorbol 12-myristate 13-acetate-induced ectodomain shedding and phosphorylation of the human meprinbeta metalloprotease. J. Biol. Chem 2003, 278, 42829–42839. 12941954 10.1074/jbc.M211169200

[21] Lewnard JA; Mora AM; Nkwocha O; Kogut K; Rauch SA; Morga N; Hernandez S; Wong MP; Huen K; Andrejko K; Prevalence and Clinical Profile of Severe Acute Respiratory Syndrome Coronavirus 2 Infection among Farmworkers, California, USA, June–November 2020. Emerg. Infect. Dis 2021, 27, 1330–1342. 33657340 10.3201/eid2705.204949 PMC8084509

[22] Mora AM; Lewnard JA; Kogut K; Rauch SA; Hernandez S; Wong MP; Huen K; Chang C; Jewell NP; Holland N; Risk Factors Associated with SARS-CoV-2 Infection Among Farmworkers in Monterey County, California. JAMA Netw. Open 2021, 4, e2124116. 34524438 10.1001/jamanetworkopen.2021.24116 PMC8444020

[23] Iwamoto C; Lesteberg KE; Lamb MM; Calvimontes DM; Guo K; Barrett BS; Mickens KL; Duca LM; Monzon J; Chard AN; High SARS-CoV-2 Seroprevalence and Rapid Neutralizing Antibody Decline among Agricultural Workers in Rural Guatemala, June 2020–March 2021. Vaccines 2022, 10, 1160. 35891324 10.3390/vaccines10071160 PMC9323551

[24] Wunschel J; Poole JA Occupational agriculture organic dust exposure and its relationship to asthma and airway inflammation in adults. J. Asthma 2016, 53, 471–477. 26785925 10.3109/02770903.2015.1116089 PMC4946797

[25] Poole JA; Wyatt TA; Oldenburg PJ; Elliott MK; West WW; Sisson JH; Von Essen SG; Romberger DJ Intranasal organic dust exposure-induced airway adaptation response marked by persistent lung inflammation and pathology in mice. Am. J. Physiol. Lung Cell. Mol. Physiol 2009, 296, L1085–L1095. 19395665 10.1152/ajplung.90622.2008 PMC2692812

[26] Yeung ML; Teng JLL; Jia L; Zhang C; Huang C; Cai JP; Zhou R; Chan KH; Zhao H; Zhu L; Soluble ACE2-mediated cell entry of SARS-CoV-2 via interaction with proteins related to the renin-angiotensin system. Cell 2021, 184, 2212–2228.e12. 33713620 10.1016/j.cell.2021.02.053 PMC7923941

[27] Zou Z; Yan Y; Shu Y; Gao R; Sun Y; Li X; Ju X; Liang Z; Liu Q; Zhao Y; Angiotensin-converting enzyme 2 protects from lethal avian influenza A H5N1 infections. Nat. Commun 2014, 5, 3594. 24800825 10.1038/ncomms4594 PMC7091848

[28] Gu H; Xie Z; Li T; Zhang S; Lai C; Zhu P; Wang K; Han L; Duan Y; Zhao Z; Angiotensin-converting enzyme 2 inhibits lung injury induced by respiratory syncytial virus. Sci. Rep 2016, 6, 19840. 26813885 10.1038/srep19840 PMC4728398

[29] Gheblawi M; Wang K; Viveiros A; Nguyen Q; Zhong JC; Turner AJ; Raizada MK; Grant MB; Oudit GY Angiotensin-Converting Enzyme 2: SARS-CoV-2 Receptor and Regulator of the Renin-Angiotensin System: Celebrating the 20th Anniversary of the Discovery of ACE2. Circ. Res 2020, 126, 1456–1474. 32264791 10.1161/CIRCRESAHA.120.317015 PMC7188049

[30] Tang N; Li D; Wang X; Sun Z Abnormal coagulation parameters are associated with poor prognosis in patients with novel coronavirus pneumonia. J. Thromb. Haemost 2020, 18, 844–847. 32073213 10.1111/jth.14768 PMC7166509

[31] Gemmati D; Bramanti B; Serino ML; Secchiero P; Zauli G; Tisato V COVID-19 and Individual Genetic Susceptibility/Receptivity: Role of ACE1/ACE2 Genes, Immunity, Inflammation and Coagulation. Might the Double X-chromosome in Females Be Protective against SARS-CoV-2 Compared to the Single X-Chromosome in Males? Int. J. Mol. Sci 2020, 21, 3474. 32423094 10.3390/ijms21103474 PMC7278991

[32] Kuba K; Imai Y; Ohto-Nakanishi T; Penninger JM Trilogy of ACE2: A peptidase in the renin-angiotensin system, a SARS receptor, and a partner for amino acid transporters. Pharmacol. Ther 2010, 128, 119–128. 20599443 10.1016/j.pharmthera.2010.06.003 PMC7112678

[33] Kuba K; Imai Y; Rao S; Gao H; Guo F; Guan B; Huan Y; Yang P; Zhang Y; Deng W; A crucial role of angiotensin converting enzyme 2 (ACE2) in SARS coronavirus-induced lung injury. Nat. Med 2005, 11, 875–879. 16007097 10.1038/nm1267 PMC7095783

[34] Zhong J; Basu R; Guo D; Chow FL; Byrns S; Schuster M; Loibner H; Wang XH; Penninger JM; Kassiri Z; Angiotensin-converting enzyme 2 suppresses pathological hypertrophy, myocardial fibrosis, and cardiac dysfunction. Circulation 2010, 122, 717–728. 20679547 10.1161/CIRCULATIONAHA.110.955369

[35] Patel VB; Zhong JC; Grant MB; Oudit GY Role of the ACE2/Angiotensin 1–7 Axis of the Renin-Angiotensin System in Heart Failure. Circ. Res 2016, 118, 1313–1326. 27081112 10.1161/CIRCRESAHA.116.307708 PMC4939482

[36] Wang K; Gheblawi M; Oudit GY Angiotensin Converting Enzyme 2: A Double-Edged Sword. Circulation 2020, 142, 426–428. 32213097 10.1161/CIRCULATIONAHA.120.047049

[37] Larsson BM; Palmberg L; Malmberg PO; Larsson K Effect of exposure to swine dust on levels of IL-8 in airway lavage fluid. Thorax 1997, 52, 638–642. 9246137 10.1136/thx.52.7.638 PMC1758613

